# Evidence of Hepatitis E Virus in Goat and Sheep Milk

**DOI:** 10.3390/v12121429

**Published:** 2020-12-12

**Authors:** Radka Dziedzinska, Miroslava Krzyzankova, Marcel Bena, Petra Vasickova

**Affiliations:** Veterinary Research Institute, Hudcova 70, 621 00 Brno, Czech Republic; dziedzinska@vri.cz (R.D.); krzyzankova@vri.cz (M.K.); bena@vri.cz (M.B.)

**Keywords:** HEV, small ruminant, milk, RT-qPCR

## Abstract

Hepatitis E virus (HEV) is the etiological agent behind hepatitis E infection. Domestic pigs and wild boars are the main animal reservoirs of HEV. Very few papers describe HEV infection in goats and sheep. As the data pertaining to the presence of HEV virus in the milk of small ruminants in Europe are lacking, the aim of this paper was to examine a representative number of milk samples from these animals. The detection of HEV genome (HEV RNA) was performed using reverse transcriptase real-time polymerase chain reaction (RT-qPCR). HEV RNA was found in 2.8% of the examined samples. Positivity ranged from 10^1^ to 10^3^ genome equivalents/mL (GE/mL) with a median of 9.99 × 10^2^ GE/mL. On the basis of these results, the milk of small ruminants could represent a source of HEV infection to consumers.

## 1. Introduction

Hepatitis E is a disease caused by infection with a virus belonging to the family *Hepeviridae*, genus *Orthohepevirus* (hepatitis E virus; HEV). Clinical signs manifest as influenza-like symptoms followed by fever, pain, emesis and signs of acute hepatitis. In most patients, it progresses asymptomatically or moderately and converts to convalescence, resulting in an underestimation of the number of cases [1,2]. A severe to fatal outcome has been reported in immunocompromised patients, especially in transplant patients [1] and pregnant women, mainly in developing countries [3]. HEV infection has at least two epidemiological profiles: (i) large outbreaks in developing countries associated with water that is contaminated with human faecal waste, and (ii) sporadic cases usually caused by the zoonotic spread of the virus from wild or domestic animals in both industrialized and developing countries. An important source is imperfectly prepared meat products and offal, usually from domestic or wild pigs [4]. The increased risk of infection to humans is also connected with the processing of meat and production of meat products through domestic pig slaughtering or direct contact with infected animals [5].

HEV strains have been found in human populations and in other animal species. Domestic pigs and wild boars are the main animal reservoirs of HEV and the species most frequently investigated [5,6,7]. HEV infection in pigs has been described all over the world, with the prevalence of antibodies against HEV ranging from 30 to 98% and HEV genome (HEV RNA) from 10 to 100% [7]. Recently, the HEV has been detected in other animal species, such as camels [8], rats [9], rabbits [10] and foxes [11]. Clinically, the infection in animals is asymptomatic, although mild hepatitis can be observed histopathologically [12]. Several papers describe HEV infection in sheep, goats or donkeys [13,14,15,16,17,18]. Most of these reports are based on serological analysis [15,17,18]; in a few, the direct detection of HEV RNA from faeces or tissues was described [14,15,18].

Knowledge of HEV within milk is limited. It has been demonstrated that HEV is present in breast milk during the acute phase of infection [19]. On the basis of the experimental infection of rhesus macaques with milk from an HEV-infected cow, it appears that milk originating from infected animals should also be considered as a new zoonotic source and may represent a risk for consumers [20]. However, studies evaluating the presence of HEV in dairy milk in Germany and Belgium were not able to detect any HEV RNA in these samples [21,22].

Data on the presence of HEV in the milk of small ruminants is even rarer. There are only a few papers that describe the detection of HEV in goat’s or sheep milk [13,16,19], but none were performed on the milk of small ruminants from European Union (EU). In addition, the number of samples in the mentioned studies were considerably limited [13,16,23].

The demonstration of HEV virus in the milk of goats and sheep in developed countries within Europe has not been performed earlier, despite the discovery of new transmission routes in developed countries being highly recommended [24].

The aim of the study was to analyse a representative number of raw milk samples from small ruminants for the presence of HEV genome. The study was carried out on a high number of milk samples and thus provides relevant information on HEV in the milk of small ruminants within the Czech Republic in the EU.

## 2. Materials and Methods

Between March and May 2019, 3612 individual samples of raw milk were obtained. The samples included 938 sheep and 2674 goat milk samples originating from 12 sheep and 128 goat farms from the Czech Republic. All farms participated in milk recording in 2019. Individual samples within each farm were mixed to obtain “pooled samples”. Each pooled sample consisted of 20 individual samples or fewer in the case of smaller herds. In large herds, multiple pooled samples were prepared. In total, 290 pooled samples were prepared: 50 from ovine milk and 240 from raw caprine milk.

From each pool sample, 2.5 mL of milk was taken for RNA isolation. Prior to the isolation itself, each pool sample was spiked with 5 × 10^6^ MS2-phage-like particles (MS2-PLP) for process control and to evaluate the efficiency of the analysis [25]. The fat layer was separated by centrifugation at 3000× *g* for 15 min at 4 °C and was removed. The sample was then acidified using 1M HCl to precipitate milk protein and viral particles (pH 3.5–4.0). Due to a higher content of proteins and minerals and thus a higher buffering capacity of sheep milk when compared to goat milk, a higher volume of HCl was necessary for ovine milk (300 µL) than for goat’s milk (200 µL). The precipitate was pelleted at 4000× *g*/10 min/4 °C, the supernatant was removed, and the pellet was resuspended in 5 mL of PBS (pH 7.2). RNA isolation was performed using 7 mL TRIreagent^®^ according to the manufacturer’s instructions (Molecular Research Center Inc., Cincinnati, OH, USA). For further detection, nucleic acid was diluted in 100 µL of RNAse free water. The limit of detection (LOD) and limit of quantification (LOQ) of the method was determined by testing decaplicates of 10-fold serially diluted MS2-PLP, which ranged from 5 × 10^7^ to 5 × 10^3^ copies per 2.5 mL of sample. LOD was specified as the lowest concentration of MS2-PLP that could be detected with a 100% probability. LOQ was defined as the smallest amount of analyte, which could be quantified with precision and accuracy under a coefficient of variation <25% [26].

The detection of HEV RNA was performed as previously described [23]. Briefly, probe-based triplex reverse transcriptase real-time polymerase chain reaction (RT-qPCR) with specific oligonucleotides (set of primers and probe) targeting two different loci of the HEV genome (highly conserved 70 nt-long sequence within overlapping parts of ORF3 and ORF2 and 113 nt-long sequence of ORF2) and an oligonucleotide set for the internal amplification control was used. The assay included RNA standards for the quantification of the target RNA and an internal amplification control RNA for the verification of the accuracy of the RT-qPCR method and for revealing false-negative results. The viral load (genomic equivalents/mL; GE/mL) was determined according to the results of the RT-qPCR and calculated extraction efficiency of MS2-PLP (recovery), as described previously [27]. Parts of the HEV ORF1 (242 nt) and ORF2 (566 nt) genes were selected for sequencing according to a previous study [28] and the HEVnet typing protocol [29], respectively.

## 3. Results and Discussion

From 290 mixed samples, eight were found to be positive for HEV RNA (2.8%). Four were from sheep and four from goat milk. Positive samples originated from seven farms; two were detected at the same farm. The positivity ranged from 10^1^ to 10^3^ GE/mL with a median of 9.99 × 10^2^ GE/mL. The highest viral load was 5.15 × 10^3^ GE/mL (Table 1). The mean efficiency of the whole analysis was 10.14% with a standard deviation of 2.32. The LOD and LOQ of the method was determined to be 2.0 × 10^3^ copies of MS2-PLP per 1 mL of goat or sheep milk.

Papers dealing with HEV and its detection in milk are rare. In Turkey, HEV was demonstrated in 18.5% of goat´s milk and 12.3% of sheep milk [13]. Testing goat´s milk in China proved the presence of HEV in all tested samples [16]. However, in both of the mentioned papers, a considerably low number of samples was tested: four samples of milk in China, and 12 and eight samples of goat and sheep milk, respectively, in Turkey. A slightly higher number of samples was included in the study from Egypt, where 280 goat milk samples were tested, and HEV RNA was demonstrated in 0.7% of cases [23]. The viral load ranged from 10^3^ IU/mL in the Egyptian study [23], to 10^4^ to 10^5^ copies/mL in the goat milk samples from China [16]. Data on HEV in milk and milk products within Europe are limited. One study focused on the detection of HEV in milk in Belgium and another in Germany. Testing more than 10% of dairy milk farms in Belgium and 400 independent dairy herds in Germany did not reveal any HEV infections [21,22]. Information on HEV in the milk of small ruminants in Europe has not been studied. In the present study, 53% and 54% of sheep and goat milk samples included in milk reporting in the Czech Republic during 2019 were examined. Testing more than half of the milk samples within one country (involved in milk recording) is therefore unique and provides representative data on the occurrence of HEV in the milk of small ruminants in middle Europe. Unfortunately, due likely to a low viral load in the positive milk samples, no specific PCR amplicons were obtained for sequencing [28,30]. Therefore, the deep characterisation of the positive samples and their comparison with previously characterised HEV strains was disabled.

The risk of HEV infection is connected with undercooked or raw meat, mainly from domestic pigs or wild boars. According to current knowledge, the consumption of milk could also be considered a potential source of infection to humans [20]. This could be of special concern, particularly in the production of traditional cheeses or other milk products made from raw milk in certain areas within Europe. Furthermore, it seems that the HEV virus is able to survive the commonly used pasteurisation temperatures and that a prolongation of pasteurisation temperatures would be needed for a sufficient virus inactivation [20]. The infectious dose of HEV, especially for the oral route of transmission, is not known and remains a topic of discussion [31]. The lowest viral load, resulting from a transfusion-acquired HEV infection, was determined to be 2 × 10^4^ IU [32]. On the basis of a dose-response oral model in pigs, 10^6^ viral particles were needed for a 50% probability of infection [33]. In our study, the viral load in the tested milk samples reached up to 10^3^ GE/mL, which in the case of the ingestion of 100 mL of milk would approach the range of an infective dose, as determined in previously published papers [32,33]. However, host susceptibility, the dose-response relationship and the virulence of different strains would also have to be considered [31]. In addition, due to the pooling of individual milk samples in this study, the viral load in individual positive milk samples could be up to 20 times higher.

The origin of the HEV found in the milk of goats and sheep is not clear. Although detailed data from the tested farms are unavailable, none of the farms also bred pigs alongside the sheep/goats. One can therefore speculate that wild animals, especially wild boars, could be a source of HEV for small ruminants. A study from 2015 showed an 18% and 23% HEV positivity for wild boars living in wild and game enclosures in the Czech Republic, respectively. In addition, HEV RNA was also detected in samples originating from red deer, roe deer and mouflons [30]. It can therefore be speculated that pastures contaminated by wild animals could be a source of infection for small ruminants.

## 4. Conclusions

In this study, we determined the presence of HEV virus in the raw milk of small ruminants in the Czech Republic. HEV RNA was detected in 2.8% of the analysed samples with a concentration ranging from 10^1^ to 10^3^ GE/mL. Even though the overall prevalence is low and further studies are needed, on the basis of these data the milk of small ruminants could represent a source of HEV infection for consumers. As more than 50% of sheep and goat herds participating in milk recording were included, the study provides representative data on the occurrence of HEV in the milk of small ruminants in middle Europe.

## Figures and Tables

**Table 1:** Detection of Hepatitis E virus in ovine and caprine milk samples.

Farm	Sheep/Goat	No. of Milked Animals	No. of Mixed Samples for Testing	No. of Mixed Positive	Viral Load (GE/mL)
Farm A	goat	454	23	1	7.68 × 10^1^
Farm B	sheep	226	12	2	5.38 × 10^2^/2.61 × 10^3^
Farm C	sheep	212	12	1	2.61 × 10^3^
Farm D	sheep	100	5	1	2.92 × 10^3^
Farm E	goat	2	1	1	1.46 × 10^3^
Farm F	goat	631	33	1	3.84 × 10^2^
Farm A	goat	11	2	1	1.54 × 10^2^

GE–genomic equivalent.
